# Formulæ for Dyspepsia

**Published:** 1890-09

**Authors:** 


### FORMULÆ FOR DYSPEPSIA.

Huchard publishes, in *Les Nouveau Remedes*, April 8, 1890, several formulæ which are claimed to be of value in the treatment of dyspepsia, especially with a view of preventing the development of flatulence. Among the remedies which the author has found most satisfactory, chloroform is the best. On account cf its irritant action, it should not be given in a state of purity or in capsules, as is so frequently done, but as chloroform water.

| | |
|---|---|
| Saturated chloroform water . . . . . | 150 parts. |
| Distilled water . . . . . . . . . . . | 120 " |
| Mint water . . . . . . . . . . . . | 30 " |

Of this mixture a tablespoonful may be taken either immediately before or during a meal.

When it is desired to employ the so-called absorbing powders, the following may be prescribed:

Powdered charcoal . . . . . . . . . . . . . ʒij.

Sodium bicarbonate . . . . . . . . . . . . ʒjss.

Calcined magnesia. . . . . . . . . . . . . ʒj.

Powdered columbo . . . . . . . . . . . . . ʒss.

Make forty powders. One powder may be taken half an hour or an hour before eating. If an antiseptic action is desired at the time of eating:

Beta-naphthol }
Salicylate of bismuth } . . . . . . . aa gr. xlv.
Magnesia }

Make thirty powders, which may be administered as above.—*American Journal of the Medical Sciences.*

